# Diet diversity and food quality score in male football players and healthy non-athlete controls in relation to oxidative stress biomarkers: a descriptive-analytical study

**DOI:** 10.1186/s13102-023-00748-7

**Published:** 2023-10-20

**Authors:** Mahsa Zare, Maede Makhtoomi, Fatemeh Mansouri, Mehran Nouri, Parvin Sarbakhsh, Mohammad Hasan Eftekhari, Bahram Pourghassem Gargari, Zainab Shateri

**Affiliations:** 1 Student Research Committee, Faculty of Nutrition and Food Science, Tabriz University of Medical Sciences, Tabriz, Iran; 2 Student Research Committee, Shiraz University of Medical Sciences, Shiraz, Iran; 3 Health Policy Research Center, Institute of Health, Shiraz University of Medical Sciences, Shiraz, Iran; 4 Department of Clinical Nutrition, School of Nutrition and Food Sciences, Shiraz University of Medical Sciences, Shiraz, Iran; 5 Department of Community Nutrition, School of Nutrition and Food Science, Shiraz University of Medical Sciences, Shiraz, Iran; 6 Department of Statistics and Epidemiology, School of Public Health, Tabriz University of Medical Sciences, Tabriz, Iran; 7 Nutrition Research Center, Department of Biochemistry and Diet Therapy, Faculty of Nutrition and Food Sciences, Tabriz University of Medical Sciences, Tabriz, Iran; 8 Student Research Committee, Ahvaz Jundishapur University of Medical Sciences, Ahvaz, Iran

**Keywords:** Dietary diversity score, Food quality score, Oxidative stress, F_2alpha_-isoprostane, 8-hydroxy-2'-deoxyguanosine, Football

## Abstract

**Background:**

Dietary patterns that include high-quality and varied food groups have the potential to modulate oxidative status. This research was conducted to determine dietary diversity score (DDS) and food quality score (FQS) in football players and their matched non-athletes, also their associations with oxidative indicators assessed by the urinary levels of F_2alpha_-isoprostane (F_2a_-IP) and 8-hydroxy-2'-deoxyguanosine (8-OHdG).

**Methods:**

Participants consisted of 45 male football players and 45 male non-athletes in two age-and body mass index (BMI)-matched groups from Shiraz City, Iran. Anthropometric measurements were performed, and urine samples were analyzed to determine oxidative biomarkers. Dietary data derived from a reliable food frequency questionnaire with 168 items was completed to determine DDS and FQS. For data analysis, an appropriate generalized estimating equation model was set up.

**Results:**

Our results demonstrated that FQS (β = 5.46; *P* < 0.001) and DDS (β = 1.30; *P* < 0.001) scores were significantly higher in the footballers in comparison to the non-athletes. Moreover, FQS was negatively associated with 8-OHdG (β=-0.35; *P* = 0.008) and F_2a_-IP (β=-4.30; *P* = 0.01) levels in all participants. In addition, DDS was inversely related to 8-OHdG (β=-1.25; *P* = 0.01) and F_2a_-IP (β=-11.67; *P* = 0.04) levels in all participants.

**Conclusions:**

Food quality scores and dietary diversity of footballers’ diets were found to be higher in comparison to the non-athletes. Furthermore, a higher FQS and DDS were associated with lower levels of oxidative biomarkers in all participants.

## Introduction

In sports, nutrition is one of the important pillars of health and performance [1]. Dietitians of Canada, the Academy of Nutrition and Dietetics, and the American College of Sports Medicine have reported that “well-chosen nutrition strategies can enhance performance and recovery from athletic activities” [2]. To investigate the relationship between diet and health outcomes, the measurement of overall diet has been seen to be more useful than the measurement of one single food or nutrient [3] due to the interactions of nutrients eating together, and their synergistic effects [4].

One of the indicators for evaluating an overall healthy diet is the dietary diversity score (DDS). A varied diet is related to more intake of macro-and micronutrients. Moreover, higher dietary diversity is a key component of higher diet quality and nutritional adequacy [5–10]. Some previous studies demonstrated that DDS is positively associated with a higher intake of all nutrients in adults [8, 11, 12]. It has been reported that DDS is inversely correlated with cardiovascular disease [13], cancers [14], and metabolic syndrome [15].

Another dietary score that considers the whole diet, the food quality score (FQS), was developed to evaluate adherence to diet quality [16–18]. FQS is calculated by summing up 14 food groups classified into two categories of healthy and unhealthy foods. Since there is no requirement for databases or software to accomplish nutrient analysis, food-based scores are suitable and easier to apply in clinical settings [16].

As an endurance-speed sport, football is characterized by highly physical and technical demanding efforts, which induce the oxidative stress (OS) process, leading to cellular damage [19]. OS is an imbalance between the production and the body’s ability to deactivate reactive oxygen species [20] and exercise training is contributed to the induction of OS by increasing the production of reactive oxygen species [21]. The muscular contraction-induced reactive oxygen species production is important in activating pathways involved in exercise-induced adaptation [22].

Two valid biomarkers of OS in clinical studies are F_2alpha_-isoprostane (F_2a_-IP) [23, 24] and 8-hydroxy-2'-deoxyguanosine (8-OHdG) [25, 26]. F_2a_-IP is an indicator of non-enzymatic oxidation of arachidonic acid, and 8-OHdG is an indicator of DNA nucleobase modifications by hydroxyl radicals [27, 28]. Studies have indicated that the urinary concentrations of F_2a_-IP and 8-OHdG are related to some factors such as diet and exercise [29].

Food intake has been reported to modulate OS [30]. Furthermore, good nutrition can influence the overall health and performance of football players [1, 31]. At the same time, previous studies indicated the need to enhance athletes’ nutritional knowledge to correct their dietary intake [32, 33].

Diversity in food intake with emphasis on fruits, vegetables, and dairy products, besides controlling the total energy intake, might be accompanied by a better body’s antioxidant status and reduced OS [34]. Furthermore, oxidative balance can be influenced by the quality of diet [3, 35]. Hence, assessing dietary diversity and dietary quality indices appeared useful in modulating oxidative status.

To our knowledge, no study exists to inspect both dietary diversity and FQS in football players, also their relationships with urinary oxidative parameters. Therefore, the current research aimed to assess DDS and FQS in football players and their matched non-athletes and investigate the association of DDS and FQS with F_2a_-IP and 8-OHdG.

## Materials and methods

### Ethical approval

This study was conducted in accordance with the ethical standards of the Declaration of Helsinki and was approved by the Ethics Committee of Tabriz University of Medical Sciences, Tabriz, Iran (under the ethical code: IR.TBZMED.REC.1399.1009), and all the subjects signed written informed consent.

### Study design, sample size, and participants

The present data was derived from a research project in which some of the findings were previously reported [19]. A total of 90 males in two groups, 45 football players and 45 healthy non-athletes, in which two groups were age-and BMI-matched, volunteered to participate in this descriptive-analytical study.

Using G-power software, the sample size was calculated based on the mean difference in urinary 8-OHdG levels between non-athlete and athlete groups following Rahimi et al.‘s study [36]. Owing to the existing correlation between study groups, the sample size of 45 subjects for each group was estimated in accordance with paired-design research by considering a 0.15 correlation between 8-OHdG levels of study groups, 95% confidence, and 80% power.

Participants were enrolled through a cluster sampling procedure. Five clubs and five schools were randomly selected among the 36 active football clubs and ten schools of Shiraz University of Medical Sciences, Shiraz City, Iran, respectively. Then, nine eligible football players and nine eligible non-athletes were recruited randomly among each selected club and school, respectively.

The inclusion criteria for athletes were as follows: (1) Football experience within the last two years with a protocol of 3–4 times/week and 90–120 min/session; (2) Aged 20–30 (years) and body mass index (BMI) 20–25 (kg/m^2^); (3) Metabolic equivalent of task (MET) > 3000 (min/week); (4) Not taking any antioxidant supplements in the last month; (5) Stable eating habits within the last two months; (6) No smoking and alcohol.

Eligible non-athletes were as follows: subjects were matched with football players according to age and BMI, 600 < MET < 3000 (min/week), items #4–6, mentioned before for the athlete group.

The exclusion criteria for both groups were as follows: (1) Consuming drugs that change the oxidants and antioxidants metabolism in the last month and nonsteroidal anti-inflammatory drugs; (2) Subjects with infectious, kidney, liver and cardiovascular diseases, hypertension, diabetes, thyroid problems, malignancies, hyperlipidemia, and stroke; (3) Those who filled out less than 90% of items of the food intake questionnaire.

### Anthropometric indices and physical activity

Anthropometric measurements and all questionnaires were completed by a skilled interviewer. A digital scale (Seca model 813, Hamburg, Germany) was utilized for the measurement of weight, and a wall-mounted stadiometer (Seca model 222, Hamburg, Germany) for the measurement of height in all participants (by considering a precision of 0.1 kg and 0.1 cm, respectively). The following formula was used to determine BMI: weight (kg)/height (m^2^). A valid short form of the international physical activity questionnaire (IPAQ) [37] was completed to determine physical activity (MET-hr/week). The InBody 270 body composition analyzer was used to estimate the fat-free mass (FFM) and fat mass (FM).

### Dietary assessment

A valid 168-item semi-quantitative food frequency questionnaire (FFQ) [38] completed via face-to-face interview by an experienced nutritionist was utilized to determine participants’ dietary intakes over the previous year. Several frequency consumption groups of each item (daily, weekly, monthly, or yearly) were converted to daily grams based on household measures [39]. Then, Nutritionist IV software (First Databank, San Bruno, CA, USA), which was modified for Iranian foods, was utilized to assess the content of energy and the nutrients of food items.

### DDS calculation

Scoring dietary diversity was calculated based on the method developed by Kant et al. [40]. It was assessed by dividing food items into five groups and 23 subgroups including: (1) Grains, including seven subgroups (biscuits, whole bread, refined bread, macaroni, corn flakes, rice, and refined flour); (2) Fruits, including two subgroups (fruit juice and fruit, citrus fruits, and berries); (3) Vegetables, including seven subgroups (starchy vegetables, yellow vegetables, potato, tomato, green vegetables, legumes, and other vegetables); (4) Meats, including four subgroups (red meat, poultry, fish, and eggs); (5) Diary, including three subgroups (cheese, milk, and yoghurt). To be considered as a ‘consumer’, the respondent had to intake at least one-half serving of each group on one day per the definitions of Food Pyramid quantity criteria. To create the total score of each main group, all subgroups’ score in each main group was summed and then divided by the number of its subgroup and, after that, multiplied by two. The scores of the main five groups were summed to calculate the overall score. Total DDS ranged from 0 to 10; that higher score represented better dietary diversity. Notably, in calculating DDS scores, the amounts of food items were adjusted for daily total energy intake using the residual method [41].

### FQS calculation

Scoring food quality was calculated using the Fung et al. study [16]. The components of FQS were classified into two food groups: (1) Healthy food groups that included nuts, vegetables, legumes, fruits, coffee, dairy, fish, and poultry; (2) Unhealthy food groups that included potato, refined grains, sugar, red meat, processed meat, solid oil, butter, and sweet chocolate. Food intakes were ranked into quintiles. Then, a score of 1–5 was allocated for each healthy food, and a reverse ranking (a score of 5 − 1) was allocated for each unhealthy food. After that, each food group’s score was summed to create the final diet score (ranging between 14 and 70) that higher scores express a healthier diet. Notably, in calculating FQS scores, the amounts of food items were adjusted for daily total energy intake using the residual method [41].

### Urinary measurements

Morning urine samples (8–10 AM) were obtained from all of the fasted participants (for athletes the three days after the last football training), and all subjects were instructed to avoid caffeine a day before urine sampling. Enzyme-linked immunosorbent assay (ELISA) was used to estimate urinary levels of 8-OHdG (8-OHdG kit, Abbexa, United Kingdom) with the catalog number of abx150312, and F_2a_-IP (8-epi PGF_2a_ kit, Abbexa, United Kingdom) with the catalog number of abx150311. Then, F_2a_-IP and 8-OHdG levels were reported based on the amount of urinary creatinine. Based on the kit instructions, the creatinine measurement was applied by spectrophotometry assay (Pars Azmoon, Tehran, Iran).

### Statistical analysis

Data were analyzed using SPSS version 25.0. (SPSS Inc., Chicago, IL, USA). The Kolmogorov-Smirnov test was used to evaluate the normality of data distribution and normally distributed variables reported by mean ± standard deviation (SD). Owing to matched design in the present study, a generalized estimating equation (GEE) by including an exchangeable correlation structure and identity link function was applied, and the significance level was 0.05 for all analyses. An appropriate GEE approach was performed to compare the mean of subject’s characteristics, nutrients and food items, and assess the differences in DDS and FQS scores in the athlete group compared with the non-athlete. Linear regression analysis in the GEE model was performed to evaluate the association of DDS and FQS scores with 8-OHdG and F_2a_-IP levels in all participants.

## Results

Table 1 outlines the values calculated for the baseline characteristics of participants. The mean ± SD age and BMI of subjects were 22.88 years (2.41) and 22.08 kg/m^2^ (1.35), respectively. Football players had significantly higher mean values of MET (hr/week), FFM (kg), DDS and FQS scores and significantly lower mean values of FM (kg), 8-OHdG (ng/mg creatinine), and F_2a_-IP (pg/mg creatinine) (P < 0.001 for all values).

**Table 1:** Baseline profile of the participants

Variables	Football players (n = 45)	Non-athletes (n = 45)	All participants (n = 90)	P-value*
BMI (kg/m^2^)	22.06 ± 1.34	22.09 ± 1.37	22.08 ± 1.35	0.90
Age (years)	22.89 ± 2.42	22.87 ± 2.42	22.88 ± 2.41	0.96
Weight (kg)	67.85 ± 7.03	66.80 ± 10.41	67.33 ± 8.85	0.47
Height (cm)	175.18 ± 6.63	173.22 ± 10.63	174.20 ± 8.86	0.29
MET (hr/week)	62.68 ± 9.04	16.21 ± 6.26	39.44 ± 24.61	**< 0.001**
FM (kg)	9.60 ± 3.12	13.10 ± 4.74	11.35 ± 4.36	**< 0.001**
FFM (kg)	58.25 ± 6.05	53.76 ± 6.98	56.00 ± 6.88	**< 0.001**
F_2a_-IP (pg/mg creatinine)	124.51 ± 41.46	241.60 ± 112.38	183.06 ± 102.76	**< 0.001**
8-OHdG (ng/mg creatinine)	10.90 ± 3.66	20.30 ± 9.52	15.60 ± 8.59	**< 0.001**
DDS	5.55 ± 1.42	4.25 ± 1.83	4.90 ± 1.76	**< 0.001**
FQS	44.56 ± 6.13	39.11 ± 6.54	41.80 ± 6.88	**< 0.001**

Variables are mean ± SD

BMI, body mass index; MET, metabolic equivalent of task; FM, fat mass; FFM, fat free mass; 8-OHdG, 8-hydroxy-2'-deoxy guanosine; F_2a_-IP, F_2alpha_-isoprostane; DDS, dietary diversity score; FQS, food quality score

*P-value is based on GEE model with identity link function and exchangeable correlation structure (study groups were matched for age and BMI)

Bold p-value (< 0.05) is considered as statistically significant

Nutrients and food groups exist in Table 2. Total energy (Kcal) (P < 0.001), protein (%Energy) (P < 0.001) and carbohydrate (%Energy) (P < 0.001) intake were significantly higher in football players, and fat intake (%Energy) was significantly greater in non-athletes. In terms of the mean of food groups: vegetables (g/day) (P = 0.01), fruits (g/day) (P = 0.009), dairy (g/day) (P < 0.001), coffee (g/day) (P < 0.001), potato (g/day) (P = 0.02), refined grains (g/day) (P = 0.009), and sweets and chocolates (g/day) (P = 0.02) were significantly higher in football players and sugars (g/day) (P = 0.02), red meats (g/day) (P < 0.001) and solid oils and butters (g/day) (P = 0.005) were significantly greater in non-athletes.

**Table 2:** Nutrients and dietary intakes of the participants

Variables	Football players (n = 45)	Non-athletes (n = 45)	All participants (n = 90)	P-value*
Energy (Kcal)	2563.12 ± 136.99	2354.31 ± 130.38	2458.72 ± 169.42	**< 0.001**
Carbohydrate (%Energy)	56.00 ± 3.35	52.75 ± 2.91	54.37 ± 3.52	**< 0.001**
Protein (%Energy)	12.65 ± 1.07	11.80 ± 1.07	12.23 ± 1.14	**< 0.001**
Fat (%Energy)	31.34 ± 3.08	35.43 ± 2.74	33.39 ± 3.55	**< 0.001**
**Food Groups**				
Vegetables (g/day)	327.04 ± 112.67	275.88 ± 97.26	301.46 ± 107.77	**0.01**
Fruits (g/day)	266.88 ± 124.21	206.71 ± 100.78	236.79 ± 116.46	**0.009**
Nuts (g/day)	23.14 ± 18.21	19.44 ± 9.76	21.29 ± 14.65	0.25
Legumes (g/day)	71.03 ± 13.30	67.19 ± 16.99	69.11 ± 15.29	0.19
Dairy (g/day)	271.70 ± 105.58	192.62 ± 80.25	232.16 ± 101.37	**< 0.001**
Coffee (g/day)	65.79 ± 10.19	15.78 ± 2.91	40.79 ± 5.89	**< 0.001**
Poultry and fish (g/day)	17.11 ± 9.56	16.99 ± 9.18	17.05 ± 9.32	0.95
Potato (g/day)	41.29 ± 11.52	35.91 ± 11.05	38.60 ± 11.55	**0.02**
Sugar (g/day)	5.33 ± 0.86	8.59 ± 0.09	6.96 ± 0.26	**0.02**
Refined grains (g/day)	461.54 ± 87.21	418.02 ± 88.95	439.78 ± 90.28	**0.009**
Red meats (g/day)	28.11 ± 9.89	40.49 ± 15.01	34.30 ± 14.09	**< 0.001**
Processed meats (g/day)	9.38 ± 2.73	8.37 ± 2.35	8.86 ± 2.54	0.34
Solid oil and butter (g/day)	6.22 ± 3.37	9.06 ± 2.37	7.64 ± 3.07	**0.005**
Sweets and chocolates (g/day)	40.23 ± 17.09	32.61 ± 14.52	36.38 ± 16.21	**0.02**

Variables are mean ± SD

*P-value is based on GEE model with identity link function and exchangeable correlation structure (study groups were matched for age and BMI)

Bold p-value (< 0.05) is considered as statistically significant

Table 3 outlines the comparison of DDS and FQS differences between the two groups. The results revealed that the football player group was greater in FQS (β = 5.46; P < 0.001) and DDS (β = 1.30; P < 0.001) compared with non-athletes.

**Table 3:** The comparison of DDS and FQS scores between football player and non-athlete groups

Variables		B	SE	95% Wald Confidence Interval (Lower, Upper)	P-value*
DDS	Non-athletes	Reference	.	.	.
	Football players	1.30	0.32	(0.67, 1.93)	**< 0.001**
FQS	Non-athletes	Reference	.	.	.
	Football players	5.46	1.23	(3.04, 7.88)	**< 0.001**

DDS, dietary diversity score; FQS, food quality score

*P-value is based on GEE model with identity link function and exchangeable correlation structure (study groups were matched for age and BMI)

Bold p-value (< 0.05) is considered as statistically significant

The linear regression findings for the relationships of DDS and FQS with 8-OHdG in all participants are available in Table 4. The findings indicated significant and negative relationships between FQS and DDS with 8-OHdG. As FQS and DDS increased by one unit, 8-OHDG decreased by 0.35 (P = 0.008) and 1.25 (P = 0.01), respectively.

Linear regression for relationships of DDS and FQS with F_2a_-IP in all participants is provided in Table 5. FOS and DDS were negatively and significantly associated with F_2a_-IP. As FQS and DDS increase by one unit, F_2a_-IP decreases by 4.30 (P = 0.01) and 11.67 (P = 0.04), respectively.

**Table 4:** The association of DDS and FQS scores with 8-OHdG level in all participants

Variables		B	SE	95% Wald Confidence Interval (Lower, Upper)	P-value*
DDS	Model 1	-1.40	0.51	(-2.41, -0.40)	**0.006**
	Model 2	-1.25	0.52	(-2.29, -0.22)	**0.01**
FQS	Model 1	-0.45	0.12	(-0.69, -0.20)	**< 0.001**
	Model 2	-0.35	0.13	(-0.62, -0.09)	**0.008**

DDS, dietary diversity score; FQS, food quality score

*P-value is based on linear regression analysis using GEE model (with identity link function and exchangeable correlation structure, and study groups were matched for age and BMI)

Model 1: Crude, Model 2: Adjusted for MET

Bold p-value (< 0.05) is considered as statistically significant

**Table 5:** The association of DDS and FQS scores with F_2a_-IP level in all participants

Variables		B	SE	95% Wald Confidence Interval (Lower, Upper)	P-value*
DDS	Model 1	-16.69	6.46	(-29.36, -4.02)	**0.01**
	Model 2	-11.67	5.84	(-23.13, -0.21)	**0.04**
FQS	Model 1	-5.47	1.63	(-8.67, -2.27)	**0.001**
	Model 2	-4.30	1.68	(-7.61, -0.99)	**0.01**

DDS, dietary diversity score; FQS, food quality score

*P-value is based on linear regression analysis using GEE model (with identity link function and exchangeable correlation structure, and study groups were matched for age and BMI)

Model 1: Crude, Model 2: Adjusted for MET

Bold p-value (< 0.05) is considered as statistically significant

## Discussion

The present study examined DDS and FQS scores and their association with urinary indicators of OS markers in male footballers and their matched non-athletes. Findings revealed that DDS and FQS scores were significantly higher in athletes than non-athletes. An inverse relationship between DDS and FQS with 8-OHdG and F_2a_-IP levels was revealed.

A healthy diet rich in fruits and vegetables and reduced total fat, saturated fat, and cholesterol can decrease OS and augment antioxidant activity [42]. One of the characteristics of a healthy diet is dietary diversity [5], which is often applied to demonstrate the quality of the diet [43]. Also, DDS positively correlates with adult dietary nutrient adequacy [44].

In the present study, the findings represented that the football players’ DDS, FQS, and FFM were significantly higher than the non-athletes. A study by Fung et al. showed that people with higher FQS tended to have higher physical activity levels [16]. Also, research by Yokoyama et al. displayed that more variety of diet was related to FFM and better physical performance in the Japanese elderly [45]. Further, a study showed that the higher the physical activity, the higher the DDS [34]. The findings of a study by Masip et al. on Finnish twins showed that people with higher dietary quality scores had more physical activity, lower BMI, and waist circumference [46]. This research found that a higher dietary quality score was associated with healthier dietary patterns, including increased fiber intake and lower total fat, sucrose, and saturated fatty acid intake [46].

Moreover, the findings indicated a negative association between DDS and urinary indicators of OS, such as 8-OHdG and F_2a_-IP. The previous studies confirm the current study’s findings. The research by Narmaki et al. indicated that the higher the DDS, the higher the blood antioxidant status. Thus, low DDS was associated with high levels of OS [34]. Moreover, a study by Kong et al. revealed that dietary diversity was related to OS reduction in older people [47]. Some studies have reported that dietary diversity is positively and significantly related to vegetable and fruit consumption [48, 49]. Also, a study by Holt et al. has shown a negative relationship between fruit and vegetable consumption with urinary F_2a_-IP [30]. Fruits and vegetables can help reduce oxidative stress due to their antioxidant compounds, including phytochemicals, which are bioactive compounds [50, 51]. Since antioxidants can repair damaged cells and destroy free radicals, increasing the consumption of fruits and vegetables containing high amounts of antioxidants is recommended to reduce oxidative stress in the body [52].

Also, the results revealed an inverse relationship between FQS and OS indicators. Research by Kim et al. illustrated that greater diet quality scores were associated with lower OS [3]. In addition, research by Kong et al. observed that a good-quality diet was related to high total antioxidant capacity [47]. Moreover, a study by Hosseini et al. indicated that vegetable, fruit, legume, and yogurt consumption was significantly greater in the highest FQS scores [53]. Asemi et al.‘s study also showed that consuming the Dietary Approaches to Stop Hypertension (DASH) diet in women with polycystic ovary syndrome for eight weeks can increase plasma levels of total antioxidant capacity and glutathione [54]. Moreover, the Hermsdorff et al. study showed that the intake of fiber and vitamin C from fruits and vegetables is related to improving oxidative stress markers in adults [55]. Vegetables and fruits are special food groups with numerous anti-inflammatory compounds and high dietary antioxidants. They contain various polyphenols, carotenoids, minerals, vitamins, and bioactive compounds [56]. In addition, a study conducted by Miller et al. presented that a healthy diet consumption rich in antioxidants in healthy people reduced urinary isoprostanes and, as a result, decreased OS [57]. In the current study, the intake of fruits, vegetables, and dairy products and FQS scores were significantly higher in athletes than non-athletes. These may justify the present study’s negative association between FQS and OS.

Exercise increases the body’s production of ROS, which can increase oxidants and cause OS [58]. OS is associated with muscle damage, fatigue, and decreased immune system function, affecting an athlete’s performance [59]. However, the effects of aerobic exercise on human antioxidant activity are controversial. Some studies have reported a reduction [60] or no change [61] in circulating antioxidants after training, while others have reported an increase in antioxidant enzyme activity [62, 63]. The present study’s findings represented the prominent role of diet in OS reduction in all study participants. Therefore, diversity and high-quality diet consumption are recommended to improve oxidative balance in athletes and physically active persons.

### Strengths and limitations

This report’s strength is its novelty, as this is the first research that has evaluated the association between dietary quality and diversity indices with urinary indicators for DNA and lipid oxidation. Moreover, food-based scores are easy-to-use tools without special analysis or databases. Therefore, they are more practical and suitable to administer in the clinical setting. As a limitation, the nature of the study limited our ability to determine the effect mechanisms of FQS and DDS on urinary indicators of OS. Additional investigations are required to confirm the current findings and explore mechanisms mediating the mentioned associations.

## Conclusions

Dietary diversity and food quality scores among footballer’s diets were found to be higher in comparison to the non-athletes. The current study showed an inverse association between dietary indices of DDS and FQS with urinary indices of OS in all participants. As a result, both DDS and FQS may help reduce OS. However, compared to FQS, DDS exerted a greater effect on OS reduction.

## Data Availability

The datasets used and/or analyzed during the current study are available from the corresponding author on reasonable request.

## Abbreviations

BMI: Body mass index
DDS: Dietary diversity score
F_2a_-IP: F_2alpha_-isoprostane
FFM: Fat free mass
FFQ: Food frequency questionnaire
FM: Fat mass
FQS: Food quality score
GEE: Generalized estimating equation
8-OHdG: 8-hydroxy-2'-deoxyguanosine
IPAQ: International physical activity questionnaire
MET: Metabolic equivalent of task
OS: Oxidative stress
